# Computerized Open-Source Navon Test (COSNaT): Normative data for the assessment of global processing abilities and simultanagnosia in the Italian population

**DOI:** 10.3758/s13428-025-02811-2

**Published:** 2025-09-12

**Authors:** Laura Veronelli, Roberta Daini, Marcello Gallucci, Carlo Toneatto, Silvia Pino, Lucia Ghielmi, Silvia Primativo

**Affiliations:** 1 Department of Psychology, University of Milano-Bicocca and Milan Center for Neuroscience (NeuroMI), Milan, Italy; 2 Department of Neurorehabilitation Sciences, Casa di Cura IGEA, Milan, Italy; 3 Optics and Optometry Research Centre, University of Milano-Bicocca (COMiB), Milan, Italy; 4 IRCCS Fondazione Don Carlo Gnocchi ONLUS, Milan, Italy; 5 Department of Psychology, University of Milano-Bicocca, Milan, Italy; 6 Department of Human Sciences, LUMSA University, Rome, Italy

**Keywords:** Navon, Normative data, Simultanagnosia, Global processing deficits, Visuo-perceptual processing, Focal attention, Equivalent scores

## Abstract

Disorders in global processing abilities may be evident when posterior cortical regions are damaged due to a focal brain lesion or neurodegenerative diseases, such as posterior cortical atrophy (PCA). While in severe cases global processing deficits are evident as simultanagnosia, subtle impairments may be present early, as in patients with multi-domain mild cognitive impairment (MCI). The aim of the present study is to provide norms for the Computerized Open-Source Navon Test (COSNaT), which has already been validated in clinical populations. After a baseline that requires identifying large and small standard letters, the test measures the ability to identify the global and local levels of incongruent Navon’s hierarchical letters. A total of 200 neurologically unimpaired participants (113 F, 56.5%) with a mean age of 57.2 years (range 20–90) and a mean education of 14.1 years (range 5–28) took part in the study. The influence of demographic variables (age, education, sex) on participants’ performance was evaluated using measures of errors and response times (global, local, and delta global/local). Raw scores, regression-based adjustments for age and education levels, and equivalent scores were provided for each index. The present study provides norms in the Italian population for the COSNaT, which has been shown to be sensitive in detecting global processing impairments and simultanagnosia in different clinical populations (PCA, amnestic MCI). The computerized test may contribute to a more accurate clinical characterization of the neuropsychological profile of patients with different etiologies, as well as in prodromal stages of neurodegenerative diseases.

## Introduction

The Navon paradigm (Navon, 1977) is a classic method for assessing the integrative abilities of local elements into a global configuration. In this paradigm, small letters (local components) are arranged to form a large letter (global component), and the task requires responding to the small or large configuration of the stimulus. Neurologically unimpaired individuals process the global level faster than the local one (Kimchi, 1992; Navon, 1977, 2003; Robertson, 1996), although this effect may reverse with increasing age (Álvarez-San Millán et al., 2022; Bouhassoun et al., 2022; Georgiou-Karistianis et al., 2006; Lux et al., 2008; Oken et al., 1999; Roux & Ceccaldi, 2001; Staudinger et al., 2011), and it is modulated by perceptual variables, with congruency, eccentricity, and size having a large effect (Rezvani et al., 2020).

From a clinical perspective, initial deficits in the global processing of hierarchical stimuli may culminate, along a continuum of progressive severity, in a profound disturbance in the simultaneous processing and integration of different elements that constitute a percept. Simultanagnosia (Coslett & Saffran, 1991; Primativo & Starrfelt, 2025; Wolpert, 1924) is a neuropsychological disorder characterized by impairment in the identification of multiple simultaneous items and the inability to integrate the global meaning of a complex scene (Duncan et al., 2003; Mazza, 2017). Patients with simultanagnosia could identify single objects but fail to integrate them into a single representation, regardless of size. In severe cases, they perceive only constituent parts of a larger object, going so far as to mistake parts of objects for whole objects, appearing “functionally blind” with severely impaired activities of daily living. Both visuo-perceptual and attentional mechanisms have been hypothesized to underlie simultanagnosia (e.g., Dalrymple et al., 2007; Thomas et al., 2012; Primativo et al., 2020; Veronelli et al., 2024; see also Neitzel et al., 2016), enhancing the difficulties in its identification and characterization.

Simultanagnosia has been associated with damage to the bilateral parieto-occipital or left occipito-temporal cortices, resulting in the dorsal and ventral variants of the deficit, respectively (Coslett & Saffran, 1991; Farah, 1990). It has been more frequently described in the context of a triad (Balint’s syndrome), including also optic ataxia and ocular apraxia (Bálint, 1995; Husain & Stein, 1988), although with dissociations between manifestations (e.g., Rizzo & Hurtig, 1987) and, in neurodegenerative diseases, it is most commonly associated with posterior cortical atrophy (PCA; da Silva et al., 2017; Crutch et al., 2017; Yoon et al., 2002).

The main aim of the present study was to provide norms for the Computerized Open-Source Navon Test (CosNAT) for the evaluation of global processing abilities, assessing the putative roles of demographic variables (age, education, sex) on performances from a large sample of Italian neurologically unimpaired participants. Regression-based adjustments and equivalent scores (Capitani & Laiacona, 1997) were provided in order to allow the comparison of performances across different tasks.

To date, there is no version of Navon’s test accompanied by normative data available in the clinical setting. Including local and global levels of processing, Navon’s hierarchical letters permit the measurement of the ability to identify multiple items simultaneously and to integrate the global meaning of a complex scene. Patients with overt simultanagnosia may be unable to identify the global letter but may be accurate with the small one. However, a computerized test that relies on indices of RTs and the number of errors might unveil slight and subtle manifestations of the disease and early deficits in global processing, along a continuum of severity. This opportunity acquires significance especially in neurodegenerative disorders involving the more posterior brain areas, the diagnosis of which is often late.

## Methods

### Participants

The study included a sample of 200 Italian healthy participants (113 females, 56.5%), right-handed at the Edinburgh Handedness Inventory (Oldfield, 1971), with a mean age of 57.2 years (SD = 21.0, range 20–90 years) and a mean education of 14.1 years (SD = 4.43; range 5–28). The sample size is in line with the results of a power analysis of 186 participants (effect size f2 = 0.06; alpha = 0.05; power = 0.80; predictors = 3) and consistent with recent procedures based on multiple regression (Capitani & Laiacona, 1997, 2017).

Participants had no history or evidence of neurological or psychiatric diseases, and/or visual disorders (e.g., cataracts not surgically removed, maculopathy). As a confirmation of normal or corrected-to-normal vision, each participant was presented with a 0.1 logMAR measured with the Milan Eye Charts (MEC; Facchin et al., 2019), placed at 3 m. Participants over 65 years old had a performance within the normal range at the Montreal Cognitive Assessment (Aiello et al., 2022; M adjusted score = 25.44; SD = 2.55). Table 1 shows the distribution of demographic data of the enrolled sample. The study was evaluated by the local commission for minimal-risk studies of the Department of Psychology of the University of Milano-Bicocca (Prot.N. RM-2023–738), and it was conducted according to the standards of the Declaration of Helsinki. All participants signed a written informed consent form before participating.

**Table 1:** Number of participants included in the study, stratified by age (years) and education (years) (sex in brackets: males + females)

	Age	20–40	41–50	51–60	61–70	71–80	81–90	Total
Education	< 8	-	-	-	-	8 (3 + 5)	7 (2 + 5)	15 (5 + 10)
	8–12	3 (2 + 1)	7 (6 + 1)	5 (4 + 1)	9 (3 + 6)	14 (4 + 10)	9 (6 + 3)	47 (25 + 22)
	13–16	21 (9 + 12)	6 (1 + 5)	14 (4 + 10)	12 (6 + 6)	8 (4 + 4)	8 (6 + 2)	69 (30 + 39)
	≥ 17	21 (9 + 12)	8 (1 + 7)	13 (7 + 6)	13 (5 + 8)	7 (1 + 6)	7 (4 + 3)	69 (27 + 42)
	Total	45 (20 + 25)	21 (8 + 13)	32 (15 + 17)	34 (14 + 20)	37 (12 + 25)	31 (18 + 13)	200 (87 + 113)

### Computerized Open-Source Navon Test (COSNaT)

The test was programmed using PsychoPy software (Peirce et al., 2019; version 2023.2.3.; https://www.psychopy.org).

**Fig. 1:**
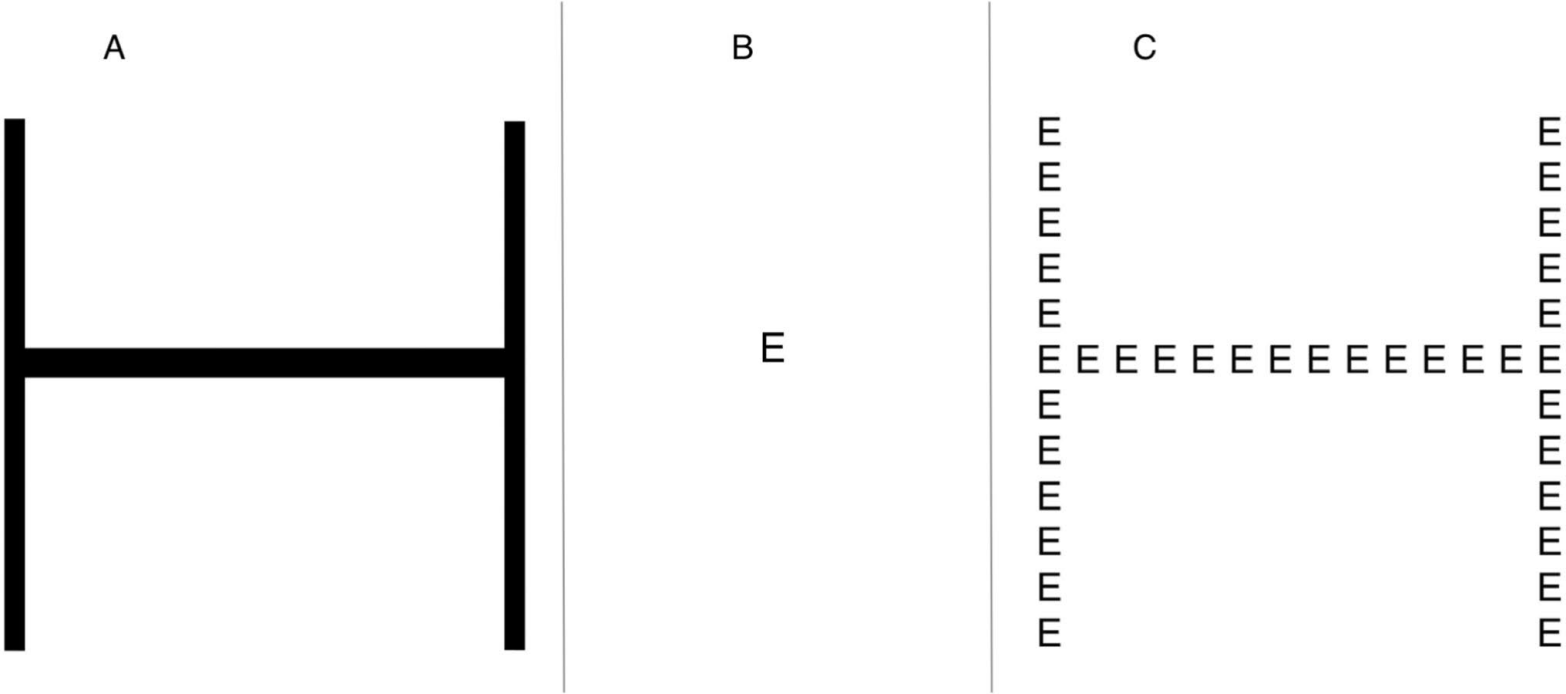
Example of stimuli used in the baseline letter identification task (panel A and B) and in the Navon task (panel C)

*Letter identification task.* The first part of the test was aimed at verifying the correct visuo-perceptual identification of big and small solid letters (Fig. 1, panel A and B, respectively). Overall stimulus’ size was 15° × 15° for the big, and 1.2° × 1.2° for the small letters. Each participant sat in front of a 13.3-inch computer monitor, with a width of 28.5 cm and a resolution of 1680 × 1050 pixels, at a viewing distance of 57 cm. Stimuli (letters H, E, S) appeared individually in the center of the screen (maximum presentation time 5000 ms): each letter was presented six times, three times in large format and three times in small format, for a total of 18 trials, following a fixed randomization. Participants were asked to press the key corresponding to the letter presented, as quickly and accurately as possible. After the response, a blank screen appeared again for 2000 ms, followed by the next trial. The response keys corresponded to the left arrow for the letter E, the down arrow for the letter H, and right arrow for the letter S. Letters were indicated on the keys to press. This response modality was chosen to avoid moving the hand on the keyboard, thus possibly improving performance accuracy. RTs and number of errors were measured.

Whether the participants show difficulty in correctly identifying solid letters, large or small (Fig. 1, panel A and B, respectively), the reliability of Navon’s test should be questioned.

*Navon task.* Stimuli consisted of global elements of three different letters (E, H, and S) formed from smaller local elements of the same letters, applying six different combinations of incongruent stimuli (Hs, He, Eh, Es, Sh, and Se), namely where the identity of the global and local letters was different (see Fig. 1, panel C). Stimulus’ size for the global and local levels corresponded to the big solid (15° × 15°) and small solid letters’ (1.2° × 1.2°) stimulus size, with an interletter distance of 0.6° (border to border).

The task consisted of four blocks, presented in a fixed ABBA order of presentation. In the first block, participants were requested to report the global or large letter, in the second block the local or small letter, in the third block the local or small letter and in the fourth one the global or big letter. Six combinations were presented in a fixed randomized order six times each, for a total of 36 trials per block, 72 trials per condition (global and local).

The first (global level) and second (local level) blocks included a practice session in which target letters were presented in random order 18 times. After three consecutive correct responses, practice was interrupted, and the task began; otherwise practice continued until the criterion was met. Presentation time and the response keys were the same as in the letter identification task.

### Statistical methods

In the single-letter identification task, participants made no errors, and therefore, no further analyses were performed. Performances of neurologically unimpaired participants at the COSNaT were analyzed using the procedure of Spinnler and Tognoni (1987) and Capitani and Laiacona (1997, 2017). Normality on raw variables was checked by evaluating skewness and kurtosis values (Kim, 2013). For the Global-, Local-, and DeltaGlobalLocal-RTs indexes, first a series of bivariate linear regression models were estimated, for each continuous covariate (age and years of education), exploring the best predicting transformation of the covariate among the linear (no transformation), natural logarithmic (Ln), base-10 logarithmic (Log_10_) of the covariate and the natural logarithm of 100 *minus* the covariate (Ln(100-x)), root square and inverse transformations. The best predicting transformation was established as the one yielding the largest R-squared, equivalent to the most effective transformation in reducing residual variance. The best predicting transformation of age and education was then included in a step-wise regression together with sex (dummy coded) in order to establish the significant predictors to condition the adjusted scores. The adjustment formula was then derived based on the coefficients of the model resulting from the step-wise regression.

The same procedure was applied to the outcome accuracy (number of errors), but due to the skewed residual distribution for accuracy data, negative binomial regressions were applied to the Global and Local number of errors. The adjustment formula was derived accordingly and expressed in the scale of the number of errors. It is worthwhile noticing that in the negative-binomial model, as opposed to the linear regression model, the predicted values are expressed in the logarithmic scale, and thus the adjustment formula requires dividing the exponential of the weighted covariates scores rather than subtracting them to obtain an adjusted score in the original outcome scale.

For the DeltaGlobalLocal-Error index, we applied the same procedure of the RTs indices using a linear regression approach because the index distribution was well approximated by a normal distribution. Importantly, calculating adjustments based on exact age and education (exact numbers of years for both variables) is more accurate than using approximations based on correction grids, as typically published in papers reporting norms. Furthermore, for Global and Local error indexes, adjustment scores based on negative binomial regressions should be divided by the raw score, instead of being subtracted as is typically the case. For these reasons, in the present paper, no correction grids with pre-calculated scores are provided, as typically done elsewhere (e.g., Banco et al., 2023; Veronelli et al., 2020), while the formulas containing the models and an Excel sheet containing the code for automatic calculation of the adjusted score for each index are made available.

Five categories were then established (equivalent scores, from 0 to 4). Zero corresponds to a score below the non-parametric one-sided 95% tolerance limit at the 95% confidence levels (the 5th observation for 200 participants) (Aiello & Depaoli, 2022); 4 corresponds to a score that is ≥ of the median value; 1, 2, and 3 are intermediate values between 0 and 4 (Facchin et al., 2022). Outer and inner non-parametric tolerance limits were also computed. Finally, percentiles were calculated based on the adjusted scores. Statistical analyses were conducted using Jamovi (version 2.6) and R software (version 4.3.3).

The script to run the test and the automated calculation sheet to obtain adjusted, equivalent, and percentile scores are freely available at the following link: https://osf.io/ankzv/?view_only=003469dbdaac4296a58f82752a4f204f

## Results

### Normative data in healthy individuals

Table 2 reports the raw scores of healthy participants in the six indexes obtained by the COSNaT, both in terms of RTs and number of errors, namely, Global-, Local-, and DeltaGlobalLocal-RTs indexes, and Global-, Local-, and DeltaGlobalLocal-Error indexes, according to the different ranges of age and years of education.

**Table 2:** Mean RTs and number of errors raw scores (SD) stratified by age and education ranges

Age	Education	Global-RTs	Local-RTs	DeltaGlobalLocal-RTs	Global-Errors	Local-Errors	DeltaGlobalLocal-Errors
20–40	< 8	-	-	-	-	-	-
	8–12	0.902 (0.199)	0.900 (0.253)	0.002 (0.062)	0.333 (0.577)	1.333 (1.155)	– 1.000 (1.000)
	13–16	0.707 (0.089)	0.706 (0.107)	0.001 (0.064)	0.524 (0.680)	0.667 (0.796)	– 0.143 (0.910)
	≥ 17	0.757 (0.229)	0.765 (0.230)	– 0.007 (0.059)	1.143 (1.682)	0.762 (1.758)	0.381 (0.921)
41–50	< 8	-	-	-	-	-	-
	8–12	0.799 (0.137)	0.783 (0.170)	0.017 (0.065)	0.429 (0.787)	0.429 (1.134)	0.000 (0.577)
	13–16	0.996 (0.116)	0.964 (0.155)	0.032 (0.081)	0.500 (0.837)	0.167 (0.408)	0.333 (1.033)
	≥ 17	0.893 (0.124)	0.841 (0.137)	0.053 (0.059)	0.125 (0.354)	0.375 (0.518)	– 0.250 (0.707)
51–60	< 8	-	-	-	-	-	-
	8–12	0.999 (0.285)	0.945 (0.264)	0.053 (0.110)	0.400 (0.894)	0.400 (0.894)	0.000 (1.414)
	13–16	1.084 (0.249)	1.002 (0.198)	0.082 (0.132)	0.500 (0.760)	0.357 (0.842)	0.143 (0.770)
	≥ 17	0.993 (0.179)	0.925 (0.175)	0.068 (0.098)	0.615 (0.768)	0.231 (0.439)	0.385 (0.650)
61–70	< 8	-	-	-	-	-	-
	8–12	1.166 (0.201)	1.027 (0.181)	0.139 (0.117)	0.222 (0.441)	0.778 (0.833)	– 0.556 (1.014)
	13–16	1.150 (0.262)	1.066 (0.189)	0.083 (0.159)	1.250 (1.865)	0.333 (0.651)	0.917 (1.917)
	≥ 17	1.127 (0.258)	1.049 (0.242)	0.078 (0.107)	0.308 (0.855)	0.231 (0.599)	0.077 (0.494)
71–80	< 8	2.020 (0.757)	1.472 (0.485)	0.548 (0.475)	5.750 (4.773)	0.875 (1.126)	4.875 (4.704)
	8–12	1.366 (0.457)	1.242 (0.456)	0.125 (0.083)	1.929 (1.774)	1.143 (2.143)	0.786 (2.607)
	13–16	1.195 (0.217)	1.049 (0.110)	0.146 (0.128)	1.875 (1.246)	0.500 (0.926)	1.375 (1.685)
	≥ 17	1.220 (0.177)	1.029 (0.142)	0.191 (0.176)	1.571 (1.272)	0.429 (0.787)	1.143 (1.676)
81–90	< 8	1.761 (0.305)	1.576 (0.299)	0.186 (0.426)	5.000 (4.123)	3.857 (6.793)	1.143 (9.529)
	8–12	1.389 (0.250)	1.213 (0.271)	0.176 (0.194)	1.000 (1.323)	0.889 (0.782)	0.111 (1.965)
	13–16	1.323 (0.296)	1.136 (0.203)	0.186 (0.244)	2.750 (3.536)	0.375 (0.518)	2.375 (3.777)
	≥ 17	1.609 (0.508)	1.220 (0.314)	0.389 (0.296)	5.000 (7.348)	1.714 (4.112)	3.286 (7.588)

### RTs indexes

For the *Global-RTs index*, bivariate linear regressions indicated that the quadratic transformation of age was the best predictor (*R*^2^ =.437). However, given the almost identical performance of the linear effect (no transformation), (*R*^2^ =.423), the linear term was selected because it is much easier to implement in the adjustment formula. The inverse transformation for years of education (*R*^2^ =.287) was selected as the best transformation for this covariate. Step-wise regression with the selected transformations and sex indicated that sex was not a significant predictor (B =.008, t_(198)_ =.189, *p* =.850). The final model showed a significant effect of age (B =.0105, t_(198)_ = 9.145, *p* <.001) and of (inverse) years of education (B = 3.777, t_(198)_ = 5.53, *p* <.001). Accordingly, the adjustment formula is:

$${Y}_{adj}={Y}_{raw}-\left[0.0105*\left(age-57.1650\right)+3.7768*\left(\frac{1}{education}-0.0809\right)\right]$$

For the *Local-RTs index*, bivariate linear regressions indicated as best predicting transformation the quadratic transformation of age (*R*^2^ =.361) and the linear (*R*^2^ =.355), which was selected for the sake of simplicity. The inverse transformation for years of education (*R*^2^ =.254) was selected as the best performing transformation. Step-wise regression with the selected transformations and sex indicated that sex was not a significant predictor (B =.018, t_(198)_ =.540, *p* =.589). The final model showed a significant effect of age (B =.0071, t_(198)_ = 7.726, *p* <.001) and of (inverse) years of education (B = 2.741, t_(198)_ = 5.010, *p* <.001). Accordingly, the adjustment formula is:

$${Y}_{adj}={Y}_{raw}-\left[0.0071*\left(age-57.1650\right)+2.74415*\left(\frac{1}{education}-0.0809\right)\right]$$

For the *DeltaGlobalLocal-RTs index*, bivariate linear regressions indicated as best predicting transformation the natural logarithmic transformation of 100 *minus* age (*R*^2^ =.191). The inverse transformation was selected for years of education because it yielded the largest *R*^2^ (*R*^2^ =.107). Step-wise regression with the selected transformations and sex indicated sex had a non-significant effect (B = –.008, t_(198)_ = –.317, *p* =.751). The final model showed a significant effect of Ln(100-age) (B = –.138, t_(198)_ = – 5.058, *p* <.001) and the inverse of education (B =.923, t_(198)_ = 2.190, *p* =.020). Accordingly, the adjustment formula is:

$${Y}_{adj}={Y}_{raw}-\left[-0.138*\left(Ln(100-age)-3.6235\right)+0.923*\left(\frac{1}{education}-0.0809\right)\right]$$

### Accuracy indexes

For the *Global-Error index*, bivariate negative binomial regressions indicated as best predicting transformation the Ln(100-x) transformation of age (*R*^2^ =.294) was the best predicting transformation, which was selected because the linear transform was not performing at a comparable level (*R*^2^ =.22). The inverse transformation for years of education (*R*^2^ =.169) was selected as the best transformation for this covariate. Step-wise negative binomial regression with the selected transformations and sex indicated that sex was not a significant predictor (B = –.274, t_(198)_ = – 1.321, *p* =.186). The final model showed a significant effect of Ln(100- age) (B = –.958, *z* = – 4.534, *p* <.001) and of (inverse) years of education (B = 6.667, *z* = 2.318, *p* =.020). Accordingly, the adjustment formula is:

$${Y}_{adj}={Y}_{raw }/ exp\left[-0.9589*\left(Ln\left(100-age\right)-3.6235\right)+6.6674*\left(\frac{1}{education}-0.0809\right)\right]$$

For the *Local-Error index*, bivariate negative binomial regressions indicated that no transformation of age significantly predicts the outcome, although Ln(100-x) transformation yielded the larger *R*^2^. The inverse transformation for years of education (*R*^2^ =.116) was selected as the best transformation for this covariate. Step-wise negative binomial regression with the selected transformations and sex indicated that neither sex (B =.042, *z* =.162, *p* =.870), nor Ln(100-age) (B = –.321, *z* = – 1.187, *p* =.235) were significant predictors of the outcome. The final model showed a significant effect of (inverse) years of education (B = 10.674, *z* = 3.230, *p* <.001). Accordingly, the adjustment formula is:

$${Y}_{adj}={Y}_{raw} / exp\left[10.674*\left(\frac{1}{education}-0.0809\right)\right]$$

For the *DeltaGlobalLocal-Error index*, bivariate linear regressions indicated that the Ln(100-x) transformation yielded the larger *R*^2^ (*R*^2^ =.06), very close to the performance of the linear transformation (*R*^2^ =.046). Thus, for the sake of simplicity, we selected the linear transformation for the age covariate. No transformation for years of education yielded a significant effect on the outcome, thus the inverse transformation was selected for the step-wise regression because it yielded the largest *R*^2^ (*R*^2^ =.03). Step-wise negative binomial regression with the selected transformations and sex indicated that neither sex (B = –.335, t_(198)_ = –.869, *p* =.385), nor education (B = 8.913, t_(198)_ = 1.387, *p* =.166) were significant predictors of the outcome. The final model showed a significant effect of age (B =.030, t_(198)_ = 3.110, *p* =.002). Accordingly, the adjustment formula is:

$${Y}_{adj}={Y}_{raw}-\left[0.0300*\left(age-57.1650\right)\right]$$

The formulas to correct each score, inner and outer tolerance limits, and equivalent scores of the COSNaT are reported in Tables 3 and 4. Percentiles of adjusted scores for the six indexes of the COSNaT are reported in Table 5. It should be noted that higher scores in the DeltaGlobalLocal-RTs and Errors must be considered indicative of worse performance.

**Table 3:** Age- and education-based adjustment formulas for the COSNaT indexes

Global-RTs
$$Adjusted\ score=Raw\ score-\left[0.011*\left(age-57.165\right)+3.777*\left(\frac{1}{education}-0.081\right)\right]$$
Local-RTs
$$Adjusted\ score=Raw\ score-\left[0.007*\left(age-57.165\right)+2.744*\left(\frac{1}{education}-0.081\right)\right]$$
DeltaGlobalLocal-RTs
$$Adjusted\ score=Raw\ score-\left[-0.138*\left(Ln \left(100-age\right) -3.624\right)+0.923*\left(\frac{1}{education}-0.081\right)\right]$$
Global-Errors
$$Adjusted\ score=Raw\ score/ exp\left[-0.959*\left(Ln \left(100-age\right) -3.624\right)+6.667*\left(\frac{1}{education}-0.081\right)\right]$$
Local-Errors
$$Adjusted\ score=Raw\ score / exp\left[10.674*\left(\frac{1}{education}-0.081\right)\right]$$
DeltaGlobalLocal-Errors
$$Adjusted\ score=Raw\ score-\left[0.0300*\left(age-57.165\right)\right]$$

**Table 4:** Equivalent scores and outer and inner tolerance limits for the six indexes of the COSNaT

	Equivalent scores					oTL	iTL
	0	1	2	3	4		
Global-RTs (sec)	≥ 2.06	2.05–1.28	1.27–1.18	1.17–1.09	< 1.09	2.07	1.50
Local-RTs (sec)	≥ 1.71	1.70–1.16	1.15–1.04	1.03–0.98	< 0.98	1.71	1.27
DeltaGlobalLocal-RTs (sec)	≥ 0.59	0.59–0.20	0.19–0.14	0.13–0.09	< 0.08	0.59	0.32
Global-Errors	≥ 5.61	5.60–2.19	2.18–1.17	1.16–0.24	< 0.24	5.61	3.32
Local-Errors	≥ 1.47	1.48–0.53	0.52–0.24	0.24–0	0	1.47	0.79
DeltaGlobalLocal-Errors	≥ 6.81	6.80–0.19	0.18 to – 0.71	–.70 to –.29	< – 1.29	6.81	1.28

**Table 5:** Percentiles of adjusted scores for the six indexes of the COSNaT

Percentile	Global-RTs	Local-RTs	DeltaGlobalLocal-RTs	Global-Errors	Local-Errors	DeltaGlobalLocal-Errors
99	2.23	1.87	0.64	6.95	3.04	8.31
95	1.63	1.4	0.37	4.25	0.96	1.89
90	1.37	1.24	0.3	2.77	0.76	0.72
85	1.31	1.18	0.22	2.31	0.54	0.34
80	1.26	1.14	0.19	2.13	0.52	– 0.15
75	1.21	1.12	0.17	1.63	0.44	– 0.45
70	1.19	1.06	0.14	1.42	0.35	– 0.66
65	1.17	1.03	0.13	1.15	0.15	– 0.72
60	1.15	1.02	0.11	0.96	0	– 0.83
55	1.12	1	0.1	0.61	0	– 1.15
50	1.09	0.98	0.09	0.21	0	– 1.3
45	1.06	0.96	0.08	0	0	– 1.53
40	1.04	0.94	0.07	0	0	– 1.63
35	1.02	0.92	0.06	0	0	– 1.74
30	0.99	0.9	0.05	0	0	– 1.89
25	0.96	0.88	0.03	0	0	– 1.99
20	0.92	0.85	0.01	0	0	– 2.24
15	0.86	0.83	– 0.01	0	0	– 2.51
10	0.81	0.78	– 0.04	0	0	– 3.31
5	0.75	0.7	– 0.12	0	0	– 4.04
4	0.73	0.69	– 0.13	0	0	– 4.04
3	0.69	0.68	– 0.19	0	0	– 4.14
2	0.63	0.63	– 0.23	0	0	– 4.49
1	0.53	0.55	– 0.29	0	0	– 4.67

## Discussion

The present study provides norms from an Italian neurologically unimpaired sample, stratified by sex, age, and education, for the Computerized Open-Source Navon Test (COSNaT), which assesses abilities of global processing using hierarchical Navon letters. The output gives six indexes: Global-, Local-, and DeltaGlobalLocal-RTs and Global-, Local-, and DeltaGlobalLocal-Errors. Age and education adjustments, equivalent scores, and percentiles are provided.

The COSNaT can be used in a clinical setting to investigate the presence of a subtle global processing deficit or simultanagnosia. Although frequently described in patients with focal brain lesions, visuo-perceptual deficits may be detectable also in the early stages of a neurodegenerative disease, as in typical Mild Cognitive Impairment (MCI; Veronelli et al., 2024), as well as in PCA as overt simultanagnosia (Primativo et al., 2020).

Diagnosis of simultanagnosia is often based on qualitative observation of patient performance in non-standardized tasks. In word reading, a patient affected by simultanagnosia may be able to spell single letters without reading the entire word. In counting dots drawn on paper, the patient might be able to count the correct number of dots only when they are connected by a line.

Very few standardized tools are available to evaluate cognitive abilities impaired in simultanagnosia. The reading test included in the cortical vision screening (CORVIST; James et al., 2001) is sensitive to revealing difficulties in the simultaneous letter perception, with spelling phenomena and letter-by-letter reading. Subtests of dot counting and number location (Visual Object Space Perception battery; Warrington & James, 1991) highlight deficits in visual-spatial organization skills, and may suggest the presence of simultanagnosia.

Typically, complex image descriptions can be used to reveal signs of simultanagnosia that become clinically evident through a patient’s inability to perceive a visual scene as a whole, while being able to perceive and recognize its individual elements. In the Cookie Theft Picture, part of the Boston Diagnostic Aphasia Examination (Goodglass et al., 2001) patients with simultanagnosia often report seeing “a boy leaning back” and “a cook” but fail to appreciate the interactions between the subjects and the overall meaning of the image. In the Picture Interpretation Test (Rosci et al., 2005), which uses a reproduction of a painting by Giacomo Favretto, exhibited at the Pinacoteca di Brera in Milan, a domestic scene is depicted, with three girls standing on chairs and a boy looking for something on the floor that is not clearly visible, but it can be guessed. The score is given by the time it takes the subject to name the word “sorcio” (mouse). In general, complex image description tests complement the clinical observation on which the diagnosis of simultanagnosia is based, but their interpretation is mostly qualitative.

Signs of simultanagnosia may also result from figure-background discrimination tests. In the Ishihara pseudoisochromatic tables, patients with simultanagnosia may show difficulty in identifying numbers despite intact abilities in color perception (Brazis et al., 1998). In the Poppelreuter-Ghent Test (Della Sala et al., 1995), they may show difficulties in recognizing each single form from the overlapping set of pictures presented.

Subtests of figure-background segregation, global vs. local processing, and shape perception are included in a computerized test that assesses perceptual organization processes, the Leuven Perceptual Organization Screening Test (L-POST; Torfs et al., 2014). In particular, we mention the embedded figure detection task, as it assesses part-whole hierarchical encoding, that is, the ability to extract a simple stimulus from a complex one by choosing among alternatives that share structurally similar features. A patient with simultanagnosia would have difficulty recognizing the target figure when it is embedded in a larger, more complex image. L-POST also includes two subtests that require selecting the name associated with an object presented in isolation or in a scene: the comparison between the two subtests could be informative for mid-level challenges encountered in real-world complex scenes where segmentation of figure and ground is required. 

Given the inclusion of two levels, the local (small letter) and the global (large letter) one, hierarchical Navon’s stimuli can be ideal to investigate disturbances in the identification of multiple simultaneous items and the inability to integrate them into a complex percept. The computerized format of the COSNaT contributes to its sensitivity in detecting markers of alterations in global processing abilities in terms of RTs and accuracy indexes, and to monitor their evolution over time.

The results of the present study showed an increase in RTs and in the number of errors with age, for both the global and local levels. Interestingly, the global precedence effect, i.e., a facilitation in global vs*.* local processing, usually found in young compared to older people, was not confirmed in our sample, with young participants processing the global and local level similarly, and the old group being faster and more accurate with the local level. The absence of a global precedence effect may be attributed to the visuo-perceptual characteristics of the stimuli used, as the large visual angle subtended by stimulus size and long stimulus duration (Bruyer & Scailquin, 2000; Rezvani et al., 2020). The parameters used in the COSNaT have been calibrated on the procedures previously validated on different clinical populations (e.g., PCA patients, Primativo et al., 2020, and MCI amnesic type, Veronelli et al., 2024). The aim of choosing a stimulus presentation that may be demanding also for neurologically healthy participants is to propose a norm-based sensitive tool to detect visuo-perceptual deficits, especially in the early stages of a progressive disease.

It worths mentioning that the design of the test includes an incongruent condition, and not a congruent one, as other studies did (e.g., Álvarez-San Millán et al., 2021). The reason for this choice is that the purpose of the study was to develop a test to detect global processing deficits in clinical settings, and the congruent condition does not, in itself, allow for the determination of which level – global or local – is processed.

Finally, norms of the present study were calculated using regressions that fit the distribution of data (e.g., applying negative binomial regressions to the Global and Local number of errors due to the skewed residual distribution of data). This methodology permits obtaining more accurate age- and education-based adjustments compared to linear models usually applied to normative data collections.

In conclusion, the COSNaT, based on proper norms on a large sample of neurologically healthy participants, can contribute to identifying global processing deficits and simultanagnosia, and to monitoring their evolution over time. This may support the early diagnosis of rarer forms of dementia, such as PCA, which are often misdiagnosed, as well as better characterize the cognitive profile in typical Alzheimer’s disease forms. Future studies will focus on providing validation data on large clinical populations with different etiologies.

## Data Availability

The study was not preregistered. Data are available on the OSF repository at the following link: https://osf.io/ankzv/?view_only=003469dbdaac4296a58f82752a4f204f.

## Code availability

The software to administer the test and the codes for adjustment score calculation are available on the OSF repository at the following link: https://osf.io/ankzv/?view_only=003469dbdaac4296a58f82752a4f204f.

The test material is licensed under a Creative Commons Attribution-NonCommercial-NoDerivatives 4.0 International License, which permits use, sharing, distribution, and reproduction in any medium or format of the original material, as long as you give appropriate credit to the original author(s) and the source, provide a link to the Creative Commons license, and indicate if changes were made. The materials may not be distributed, whether modified, transformed, or used for commercial purposes. The images or other third-party material in this article are included in the article’s Creative Commons license, unless indicated otherwise in a credit line to the material. If material is not included in the article’s Creative Commons license and your intended use is not permitted by statutory regulation or exceeds the permitted use, you will need to obtain permission directly from the copyright holder. To view a copy of this license, visit https://creativecommons.org/licenses/by-nc-nd/4.0/.

PsychoPy is licensed under the GNU General Public License (version 3). For the complete text of that license, see https://www.gnu.org/licenses/gpl-3.0.html. In basic terms, this allows you to use, redistribute, add to, modify, or even sell PsychoPy or any of its components. If you do release any such ‘derivative works’, however, you must make this source code available with the distribution and must also license your product under the GNU GPL.
